# Feasibility and impact of a post–discharge geriatric evaluation and management service for patients from residential care: the Residential Care Intervention Program in the Elderly (RECIPE)

**DOI:** 10.1186/1471-2318-14-48

**Published:** 2014-04-16

**Authors:** Penelope Harvey, Meg Storer, David John Berlowitz, Bruce Jackson, Anastasia Hutchinson, Wen Kwang Lim

**Affiliations:** 1 Department of Medicine and Aged Care, Northern Health Melbourne, Melbourne, Australia; 2 Aged Care Austin Health, Melbourne, Australia; 3 Institute for Breathing and Sleep, Austin Health, Melbourne, Australia; 4 Monash Medical Centre, Monash University, Melbourne, Australia; 5 Northern Clinical Research Centre, Northern Health & School of Nursing & Midwifery, Deakin University, Melbourne, Australia; 6 Department of Medicine and Aged Care, Northern Health & Department of Medicine, The University of Melbourne, Melbourne, Australia

**Keywords:** Residential aged care facilities, Geriatric assessment, Advance care plans, Acute health care utilisation

## Abstract

**Background:**

Geriatric evaluation and management has become standard care for community dwelling older adults following an acute admission to hospital. It is unclear whether this approach is beneficial for the frailest older adults living in permanent residential care. This study was undertaken to evaluate (1) the feasibility and consumer satisfaction with a geriatrician-led supported discharge service for older adults living in residential care facilities (RCF) and (2) its impact on the uptake of Advanced Care Planning (ACP) and acute health care service utilisation.

**Methods:**

In 2002–4 a randomised controlled trial was conducted in Melbourne, Australia comparing the geriatrician–led outreach service to usual care for RCF residents. Patients were recruited during their acute hospital stay and followed up at the RCF for six months. The intervention group received a post-discharge home visit within 96 hours, at which a comprehensive geriatric assessment was performed and a care plan developed. Participants and their families were also offered further meetings to discuss ACPs and document Advanced Directives (AD). Additional reviews were made available for assessment and management of intercurrent illness within the RCF. Consumer satisfaction was surveyed using a postal questionnaire.

**Results:**

The study included 116 participants (57 intervention and 59 controls) with comparable baseline characteristics. The service was well received by consumers demonstrated by higher satisfaction with care in the intervention group compared to controls (95% versus 58%, p = 0.006).

AD were completed by 67% of participants/proxy decision makers in the intervention group compared to 13% of RCF residents prior to service commencement. At six months there was a significant reduction in outpatient visits (intervention 21 (37%) versus controls 45 (76%), (p < 0.001), but no difference in readmissions rates (39% intervention versus 34% control, p = 0.6). There was a trend towards reduced hospital bed-day utilisation (intervention 271 versus controls 372 days).

**Conclusion:**

It is feasible to provide a supported discharge service that includes geriatrician assessment and care planning within a RCF. By expanding the service there is the potential for acute health care cost savings by decreasing the demand for outpatient consultation and further reducing acute care bed-days.

## Background

The demography of the Australian population is changing resulting in a greater need for the health care system to develop innovative models of care that will meet the complex needs of frail, older adults in a community setting [1,2]. The correlation between increasing age and reduction in functional ability means that older adults are large consumers of acute hospital care [3,4] and commonly have longer lengths of stay than younger persons [5]. Evidence suggests that when admitted frail older adults are at an increased risk of adverse effects, such as physical deconditioning, functional decline, pressure injuries, malnutrition, falls and acute delirium [6-8]. Changing health care policies, together with closure of hospital beds and shortened length of stay have seen a trend towards providing older people with more specialised care in a community setting [1,5]. Services that provide comprehensive geriatric assessment and subsequent care in the community are one alternative to sending older adults to hospital [9]. This approach has been demonstrated to be highly effective for community-dwelling older adults [9], however there is less evidence surrounding its effectiveness for those in long-term residential care facilities (RCF).

As more people enter long-term institutional care; innovative models of medical service delivery will be imperative, to promote best practice for residents whilst containing healthcare costs [2]. There is considerable international interest in alternative models of care for long-term RCF residents [10,11]. The Netherlands has successfully trialled the use of RCF physicians with specialist training in nursing home medicine and have demonstrated improvements in the quality of care provided within RCF [10]. In the US interventions to up-skill RCF staff in the assessment and management of acute inter-current illness have reduced acute care transfers from RCF facilities [11]. These successes demonstrate that medical management of RCF patients can be improved, placing the challenge on local health care administrators to develop models of care that are efficacious and applicable to their local context.

The proportion of adults dying within residential aged care facilities (RCF) is also rising. In the United States (US) it is estimated that 67% of residents will die within their facility [12]. These trends highlight the need to promote Advance Care Planning (ACP) and documentation of Advance directives (AD) within RCF and to up-skill residential care staff in the provision of palliative care [13-16]. When ACP discussions are backed up by formal documentation of AD this can facilitate decision making at a future crisis point, easing the burden on family, and care providers [13]. Despite the willingness of older adults to discuss their preferences for end-of-life care and the benefits of formally documented AD, its uptake in RCF has been relatively low [17,18].

The Residential Care Intervention Program in the Elderly (RECIPE) service is based in outer metropolitan Melbourne, Australia and provides expert comprehensive assessment and management by geriatricians and aged care nurse specialists to individuals living in RCF who are at imminent risk of requiring acute care management. In 2002 the hospital aged care unit established the service and promoted it to RCFs and general practitioners (GPs) in their catchment area. At this time, local RCFs had limited access to primary care physicians, ACP was not widely promoted in RCF and there were few alternatives to ED attendance for management of acute illness outside standard office hours. Prior to commencement of the service RCF staff reported that approximately 13% of residents had formal AD and that there was no formal system for documenting or communicating this amongst care providers.

The aims of the RECIPE service are to improve residents’ quality of life by providing them with optimal medical care within the facility, increase opportunities to discuss ACP and document ADs, promote greater consumer engagement in their care, and to improve communication between RCF and acute care clinicians. It was anticipated that if these aims were achieved then emergency department attendances would also be decreased. When the service was established, a comprehensive health service evaluation was undertaken to evaluate the feasibility, acceptability to consumers, and the potential of this model of care to decrease acute health care utilisation. This paper presents the findings of a preliminary study which evaluated both the feasibility of the geriatrician-led, in-reach service and of conducting a randomised controlled trial (RCT) to evaluate this model of care [19,20].

## Methods

### Study design

In 2002–04 we conducted a randomised controlled trial of a supported discharge intervention for patients aged 65 years or older admitted to hospital from RCF in outer metropolitan Melbourne, Australia. To efficiently utilise limited resources the patient rather than the facility was chosen as the unit of randomisation. Patients being discharged to RCF were invited to participate during their index hospital admission and were followed for six-months. Patients were excluded if they were less than 65 years of age, were not living permanently in RCF, had already been enrolled, had non-medical primary diagnoses, were expected to die during their index admission, lived outside the health service catchment area, exhibited severe behavioural disturbance, or consent was not obtained for study participation. Patients were randomised in a 1:1 ratio using a computer generated random number sequence and study allocations were placed in pre-numbered, sealed envelopes. The study team allocated each patient to the next consecutive number at discharge from acute care. They had no control over the timing of discharges, and the treating medical units were blinded to the study allocation.

Ethics approval was obtained from the Northern Health Human Research Ethics Committee and written informed consent was obtained from competent patients or the person responsible if the patient lacked capacity.

#### Intervention

The RECIPE team comprised two part-time geriatricians and an aged care nurse consultant. All intervention group patients were reviewed in the RCF within four days of discharge. At the first visit, a comprehensive assessment and a tailored care plan was developed. Appropriate services were provided and patients were offered further visits for review of intercurrent illness if required. The service also provided education and support to RCF staff and the patients’ primary care physician.

#### Usual care

The usual care group was managed by the treating medical unit according to standard hospital protocols and received standard discharge planning, with follow-up at the RCF by their primary care physician service.

#### Study assessments

Baseline demographics, medical history, current medications, quality of life was measured using the Quality of Life-Alzheimer’s Disease (QOL-AD) instrument [21], and cognition was assessed using the Abbreviated Mental Test Score (AMTS) [22] or Mini Mental State Examination (MMSE) [22]. Barthel Index [23] was used to measure physical function, and Short Zung Interviewer-assisted Depression Scale [24] was used to assess mood. Both groups were visited by the research team three times over six months for data collection. The Barthel Index, the number of medications and number of co-morbidities were used as proxy measures of illness severity and frailty amongst residents.

#### Outcomes

Data was collected on the proportion of patients and/their families who participated in advanced care planning discussions, the number of meetings that took place, the proportion who chose to document an AD and their stated preferences for end of life care. Administrative data was obtained on utilisation of hospital-based clinical services including: inpatient admission (acute or sub-acute), outpatient and day procedure visits.

#### Consumer feedback

Surveys were distributed to patients (where appropriate) and family members from both intervention and control groups. Participants were asked to provide feedback on their satisfaction with the service and whether geriatrician-led care in the RCF provided an alternative to hospital-based care. A staff feedback survey was distributed to RCF staff and the patients’ primary care physicians who were asked to provide feedback regarding service quality and effectiveness, (specifically whether the RECIPE in-reach program provided a viable alternative to ED transfer for assessment and management of intercurrent illness). The surveys were distributed with a stamped, self-addressed envelope within four weeks of the six month discharge visit, or death.

#### Statistical analysis

Statistical analysis was performed using the Statistical Package for Social Scientists (SPSS; Chicago, Illinois, version 11.0) on an intention to treat basis. Categorical data was summarised using means and percentages and continuous data using mean, standard deviation (SD) and range. Categorical outcomes were compared between groups using chi square tests and logistic regression and continuous variables were compared using independent sample t-tests. Tests of significance were two-tailed, using a significance level of p ≤ 0.05.

### Sample size

We estimated that 550 subjects were needed to detect a 10% difference in acute care readmission rates at 80% power, and an alpha level of 0.05. An interim analysis of the study results was conducted at 18 months to review the feasibility of the service and the appropriateness of the evaluation strategy. At this time point 123 patients had been randomised into the study.

### Feasibility

The feasibility of the service was measured by: consumer feedback regarding the acceptability of the service, improvements in residents’ quality of life and functional outcomes, uptake of opportunities to discuss ACP and document AD and changes in acute health care utilisation. The appropriateness of the study design was measured by assessing study recruitment rates and reasons for non-enrolment and the sensitivity of the study outcome measures to detect changes in residents’ health status.

## Results

### Study recruitment

**Figure 1:**
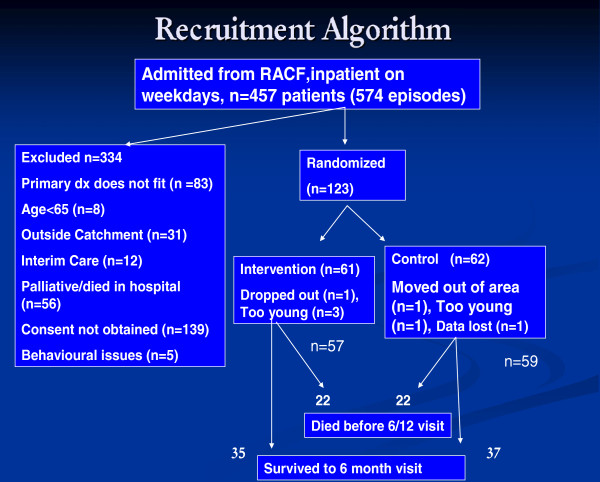
Flow diagram of patients included in this analysis.

Over 18 months, 457 patients were screened and 334 excluded. 123 patients were included in the study (Figure 1), 37% of patients in each group were male. Comparing included and excluded patients: the mean age was 84 (SD 8) years versus 82 (SD 10) years; 63% versus 54% were born in Australia; and 72% versus 65% spoke English as their primary language.

### Baseline characteristics

Of the 123 study participants, 57 patients were randomised to the intervention group and 59 patients to usual care. Participants were spread across 45 facilities and primary care was provided by more than 60 GPs. Intervention group patients were younger (mean age 83.8 vs 86.7 years, p = 0.02) and had more comorbidities than controls (mean number 7.7 vs 5.7, p < 0.001), (Table 1). *Marked dependence* occurred in 42% of controls versus 32% of intervention patients, and there was no significant change in functional scores in either group over time. At baseline 33(58%) of the intervention group and 35(59%) of controls completed the QOL-AD instrument. Due to increasing frailty and the high mortality rate, only 93% repeated the questionnaire at one month and 66% at six months. There were no significant differences between groups in quality of life at baseline and no significant changes within either group over time.

**Table 1:** Baseline characteristics

**Demographics**	**Intervention (n = 57)**	**Control (n = 59)**	**Significance (p value)**
Age (Mean, SD) years	83.8 (7)	86.7 ( 7)	0.02
Male n (%)	19 (33)	24 (41)	0.45
Low-level RACF n (%)	27 (47)	26 (44)	0.85
Australian born n (%)	34 (60)	38 (64)	0.43
English speaking (%)	44 (77)	45 (76)	0.66
Severe Dementia			
AMTS** < 4/10	47%	50%	
Depression			
(> 70 Zung Depression Scale)	7%	3%	0.40
**Index admission**			
**Primary reason** n (%)			
Pneumonia	18 (31)	16 (27)	NS
Urinary tract infection	7 (12)	8 (14)	NS
Heart failure	7 (12)	4 (7)	NS
Anaemia	5 (9)	7 (12)	NS
Volume depletion	6 (10)	6 (10)	NS
Cellulitis	2 (4)	4 (7)	NS
Chest pain	1 (2)	7 (12)	NS
Cerebrovascular event	3 (5)	2 (3)	NS
Chronic obstructive pulmonary disease	2 (4)	1 (2)	NS
Other	6 (11)	4 (7)	NS
Number of medications (mean, SD)+	10.4 (4.4)	8.5 (3.9)	0.05
Number of co-morbidities (mean, SD)#	7.7 (2.7)	5.7 (2.5)	<0.001
Index length of stay*			
(mean, SD)	10.1 (12.6)	12.1 (15.7)	
(Median, IQR)	6.0 (4-10)	7.0 (4-13)	0.53
**No of follow-up visits (mean number per patient)**			
Standard visits (study data collection)	2.4	2.6	NS
Family meetings	1.2	nil	
Allied health referrals	0.6	nil	
Hospital in the home referrals	0.5	nil	
Palliative care referrals	0.2	nil	
**Total number of study reviews & RACF visits**	**16.7**	**5.2**	NS

**Abbreviated Mental Test Score (AMTS) or Mini Mental State Examination (MMSE).

+ Number of regular and as required medications listed on medication discharge list printed by hospital pharmacy.

*Number of days spent in hospital during the admission for which the patient was invited to participate, including acute and sub acute bed-days.

#Combined number of active medical problems listed on medical admission notes and discharge summaries.

### Mortality

The overall six-month mortality was 38% with no difference between groups; (controls (37%) versus intervention (39%), (p > 0.05). In the control group 11(50%) of deaths occurred within one month of randomisation, compared with 7(32%) of deaths in the intervention group, p = 0.20. There was no significant difference in age, hospital length of stay, number of medications, co-morbidities, depression scores or study group between survivors and non-survivors. Survivors had better cognition scores (mean MMSE 13.4 versus 6.4, p = 0.01) and better functional status that those who died (mean Barthel score 48 versus 34, p = 0.04).

### Participation in advance care planning and advance directive completion

Of the 54(95%) intervention group residents who survived to the first post-discharge visit and could be offered an ACP discussion, 41(76%) residents and their families participated in at least one family meeting and ACP discussion and 36(67%) documented an AD. The mean time from enrolment in the service to written AD documentation was 40 days, (range 0 to 184). Only 8% of residents completed an AD themselves, the majority being documented by legally appointed proxy decision makers (23%) or family members (69%). Of those with a completed AD 56% avoided a subsequent hospital admission, and 44% were readmitted at least once. Of those with completed ADs, 78% of residents/proxies preferred to receive acute treatment for medical deteriorations within the facility however, 42% also considered acute care admission to be a reasonable option. Most participants did not want aggressive medical treatment including cardiopulmonary resuscitation (CPR) (70%). However 83% wanted to have initial medical investigations performed and to receive limited active treatment (eg: blood tests and intravenous antibiotics) for reversible conditions such as acute infection.

### Consumer feedback

Resident/family satisfaction surveys were distributed to 78% of participants with a response rate of 49%. The majority (80%) of the intervention group (compared with 45% of controls) had the subjective impression that the RECIPE service had successfully helped to avoid hospital readmission (Table 2). The GP survey response rate was 49% with the majority caring for intervention group patients. In this group 65% found the service useful, 75% agreed that RECIPE was an attractive alternative to hospitalisation and 70% reported that residents were less likely to be sent to hospital.

**Table 2:** Consumer feed-back - family/resident satisfaction with the RECIPE service versus usual care

**Proportion satisfied with the service they received**	**Intervention n = 20 n (%)**	**Controln = 24 n (%)**	**Significance**
Medical assessment & treatment	19 (95%)	10 (42%)	<0.001
Education	18 (90%)	13 (54%)	0.018
Level of care assessment	10 (50%)	7 (29%)	NS
General Assessment	18 (90%)	15 (63%)	0.044
Advice/phone contact	19 (95%)	10 (42%)	<0.001
Purchase of services	11 (55%)	7 (29%)	NS
Coordination of care	12 (60%)	5 (21%)	0.013
Development of care-plan	18 (90%)	9 (38%)	0.002
Advanced care planning	17 (85%)	9 (38%)	0.002
Patient review	19 (95%)	15 (63%)	0.013
Family discussion	19 (95%)	12 (50%)	0.002
Response times	16 (80%)	9 (38%)	0.006
Overall satisfaction*	**19 (95%)**	**14 (58%)**	**0.006**

*Highly satisfied = number of respondents selecting “useful or very useful” or “satisfied or very satisfied” and for response times “very good or excellent”.

### Service feasibility

Patient recruitment was slower than expected mainly due to difficulties in obtaining written informed consent. There were high numbers of eligible patients who were frail and cognitively impaired and their family members were reluctant to consent for research that might cause increased fatigue or anxiety. Recruitment was slower at the start of the study and improved as RACF staff and GPs become familiar with the service and could appreciate its potential benefits. Carers were happier to provide consent if they knew there would be an additional service, rather than taking a 50% chance that they would get “nothing”. By the end of the recruitment period family and facility staff were highly supportive of the service appearing disappointed when a resident was allocated to the control group. This positive feedback was reflected in a high retention rate after randomisation and consumer feedback that indicated that the benefits of participation outweighed the costs.

Due to slower than anticipated recruitment rates and high mortality rates the study was underpowered to demonstrate an impact on acute healthcare readmission rates. Over the six-month follow-up, there were a total of 28 ED presentations from controls and 19 from the intervention group (p = 0.4). The overall readmission rate was 36%, (intervention group 22/57 (39%) versus controls 20/59 (34%), (Table 3). Factors predictive of readmission were: length of stay at the index admission (readmitted 9 days versus not readmitted 12 days, p = 0.02) and the number of medications on recruitment (readmitted 11 versus not readmitted 9, p = 0.03). Control group patients used 372 bed-days over six months versus 271 bed-days by the intervention group (Table 2). An unexpected finding was however that rapid access to geriatrician review in the RCF did have an impact on the number of hospital ambulatory care visits. Intervention group patients 21/57 (37%) were significantly less likely to need to attend medical outpatient clinics than controls 45/59 (76%), (p < 0.001).

**Table 3:** Hospital utilisation

**Number of readmissions**	**All patients N = 116**	**Intervention**	**Control**	**Significance**
Acute Care (N)	55	29	26	0.47
(Mean, SD)/patient	0.47 (0.77)	0.51 (0.76)	0.44 (0.79)	0.60
Sub-acute care (N)	10	4	6	0.75
(Mean, SD)/patient	0.09 (0.31)	0.07 (0.26)	0.10 (0.36)	0.60
Total (N)	65	33	32	0.61
(Mean, SD)/patient	0.56 (0.89)	0.58 (0.84)	0.54 (0.93)	0.80
**Hospital Bed-days**				
Index LOS (total days)	1289	573	716	
(Mean, (SD)/patient)		10.0 (12.6)	12.1 (15.7)	0.43
Acute Care Readmit (total days)	388	193	195	
(Mean, (SD)/patient)		3.4 (6.5)	3.3 (6.9)	0.95
Sub-acute Readmit (total days)	255	78	177	
(Mean, (SD)/patient)		1.4 (5.5)	3.0 (14.7)	0.43
**Total Bed-days over follow-up**	**643**	**271**	**372**	
(Mean, (SD)/patient)		**4.8 (9.2)**	**6.3 (17.2)**	**0.55**
**Total bed days:**	**1932**	**844**	**1088**	
**index + readmission**		**14.8 (16.2)**	**18.4 (22.4)**	**0.32**

## Discussion

This study has demonstrated that it is feasible and acceptable to provide a geriatrician consultation service for people living in long-term RCF. The key strengths of the RECIPE service were that the comprehensive assessment and care planning intervention identified key ongoing medical issues enabling implementation of better management plans and improving communication between consumers, RCF staff, GPs and the acute-care based aged care team. Rapid access to geriatrician review for assessment of intercurrent illness also had a significant impact on the demand for outpatient specialist review, and may provide one mechanism to address burgeoning demand on hospital ambulatory care services.

Although the cost of providing specialist outpatient clinics is not high per consultation, the demand for such services is growing, resulting in long waiting times to obtain appointments and considerable expense and inconvenience to consumers, particularly arranging transport from RCFs to attend appointments. In contrast to patients who are reviewed by multiple specialists [25], there is evidence that review by a single specialist whose primary discipline is managing multiple comorbidities in frail, elderly clients may result in lower medication prescription rates and lower numbers of referrals for invasive procedures [10]. This will help contain health care costs and result in lower levels of inconvenience to patients and their families. It is reassuring to note that acute care readmissions and mortality were equivalent between the two groups indicating that decreasing the number of specialist outpatient reviews and managing acute medical deteriorations within RCFs is a viable alternative to acute care admission and does not place patients at risk of worse clinical outcomes.

Although the main focus of the intervention was providing alternatives to future acute care admission, the high six-month mortality rate highlighted the need for advanced care planning discussions and the provision of end of life care within the RCF. We found that a proactive program that promoted and facilitated ACP discussions and documentation of AD in RCF was well accepted by patients, and their proxies/families. This aspect of the intervention appears to have addressed an important gap in communication around end of life care and provision of palliative care services within RCF [26]. Despite this a number of barriers remain to be addressed before this practice becomes widespread [27,28]. Since this preliminary study was completed in 2004, evidence-based guidelines for the implementation of a palliative approach in Australian RCFs have been published and there is active promotion of programs to up-skill RCF staff in the provision of palliative care [29]. The RECIPE service used geriatricians to conduct family meetings and ACP discussions with patients and families, one of the challenges with this model is the cost of providing this level of expertise to all RCFs particularly those in rural and remote regions of Australia. Further work is needed to evaluate whether primary care physicians or advanced practice aged care nurses [30] can be used to promote the widespread uptake of ACP in RCF across Australia.

### Strengths and weakness of the study

We found that using a rigorous evaluation methodology that included input from key consumers helped to identify important issues in service design and evaluation that needed to be addressed prior to rolling out a larger–scale program and associated evaluation project [19]. The number of patients screened for eligibility over an 18-month period was smaller than expected, possibly because retrospective administrative data overestimated the number of permanent RCF residents in our catchment and because screening was only conducted during office hours. Despite the identified barriers to recruitment, the enrolment rate in this study was higher than that previously reported in RCF, possibly related to the inclusion of patients requiring palliative care [31]. Recruitment improved as RCF staff and GPs become familiar with the service and could appreciate and promote its potential benefits. This was reflected in our high retention rate after randomisation and consumer feedback that indicated that the benefits of participation outweighed the costs [32]. This study was underpowered to demonstrate an impact on acute care readmission rates and the lower than anticipated recruitment rates indicates that a multi-site study would be required to achieve adequate patient numbers to achieve statistical power within in a reasonable timeframe.

The Barthel Index, the number of medications and co-morbidities were used as proxy measures of illness severity amongst residents, however these measures were not appropriate once patients entered the palliative stage of their illness. In future studies measures such as the Cumulative Illness Rating Scale for Geriatrics [33] may be able to discriminate more accurately different levels of illness severity and hospitalisation risk in the RCF population. The measures of patient reported outcomes (PROs) chosen for this study were selected as they were validated, sensitive to change and brief [21,31], despite this the majority of patients struggled to complete all questionnaires at one visit. Similar to previous studies [31] the high prevalence of cognitive impairment meant that PROs were only assessed in a sub-group of participants and in the subgroup of patients who completed these measures there were no significant changes over time. Given the frailty of participants in this study and the number who later required supportive and palliative care it appears that in future studies shorter follow-up times (to increase completion rates at follow-up) and alternative measures of the quality of supportive care provision, may be more appropriate [19,32,34]. As recommended in the MORECare statement on studies evaluating complex interventions in end of life care [19] the use of a mixed methods approach that includes qualitative data obtained from consumer interviews or focus groups may be a richer source of consumer input than the written survey responses that were used in this study.

## Conclusion

This study has demonstrated that it is feasible and acceptable to provide a post-discharge outreach service to frail older people living in RCFs. The hypothesis that a post discharge, multidisciplinary assessment and management program could reduce readmissions and improve quality of life could not be proven, in part related to the unexpectedly small sample size. Although the main focus of our study intervention was comprehensive geriatric assessment and chronic disease management, it became clear that there was also a need for advanced care planning and palliative care support in RCFs in Australia. Consent for entry into randomised controlled trials will remain one of the difficult issues, a cluster randomised controlled trial of aged care facilities rather than individuals may be one way to overcome this barrier to recruitment in future studies [31].

Our results indicate that a multi-faceted approach is required to have a substantial impact on acute care readmissions rates. One alternative to a patient–level intervention would be a cluster randomised controlled trial of a facility level intervention focusing on staff education and skills and raising awareness of residents and their families regarding treatment and care options [29,30]. Strategies that would need to be incorporated into this approach include: up-skilling of RCF staff in management of acute deteriorations [12], increased access to telehealth consultations as an alternative to emergency department attendance when primary care physicians are unavailable for consultation and increased use of ‘Hospital in the Home’ services [10,11]. Since completion of this initial evaluation the RECIPE service has been extended to include these components and further evaluation of its impact on acute care utilisation over the longer–term is currently being undertaken with a larger sample size of patients.

## Abbreviations

AMTS: Abbreviated mental test score; ACP: Advance care plan (planning); CPR: Cardiopulmonary resuscitation; ED: Emergency department; GP: General practitioner; MMSE: Mini mental state examination; QOL-AD: Quality of life in Alzheimer’s disease; RCF: Residential care facility; RECIPE: Residential care intervention program in the elderly; SD: Standard deviation.

## Competing interests

The authors have no competing interests to declare.

## Authors’ contributions

PH was involved in study design, implementation of the intervention, data collection, management & analysis and preparation of the manuscript; MS was involved in implementation of the intervention and data collection; DJB, BJ & WKL contributed to study design, supervision of the clinical team, data analysis and manuscript preparation; AH was involved in data analysis, interpretation of study findings & manuscript preparation. All authors have read and approved the final manuscript.

## Pre-publication history

The pre-publication history for this paper can be accessed here:

http://www.biomedcentral.com/1471-2318/14/48/prepub
